# Charge to the Graduating Class, Delivered at the Recent Commencement in the Medical Department of the University of Buffalo

**Published:** 1867-04

**Authors:** James P. White


BUFFALO

### Medical and Surgical Journal.

VOL. VI. APRIL, 1867. No. 9.

### Original Communications.

ART. I.—*Charge to the Graduating Class, delivered at the recent Commencement in the Medical Department of the University of Buffalo.* BY PROF. JAMES P. WHITE.

GENTLEMEN:—Your educational curriculum is fulfilled. Your pupilage and your direct connection with the University terminates with the conferring of these degrees; and you now stand certified of fitness for duties of a more active and responsible character. Our daily meetings are at an end, and it but remains for us to say, farewell. You are now about to embark on the untried ocean of life. As the sails are unfurled to the breeze, it is customary and proper for some one familiar with the voyage, as well as may be, to delineate the waters which are to be traversed, point out the stars which are to guide the young mariner safely on the passage, and mark some of the rocks and quicksands upon which shipwreck is most imminent. By the partiality of my colleagues I am commissioned to address you upon this occasion. Would that it had fallen to abler hands; for this last counsel should possess the value of moral and religious excellence; and like the closing notes of a musical strain, this last impression should leave a pleasing echo on the ear. As the ancients threw their letters into the funeral pile that their departed friends might, even in the land of spirits, read them, so throw we as our last offering these hasty words of warning and exhortation after you, for your consideration when tossed to and fro by the waves of this new life upon which you are just embarking.

In the fulfillment of this duty nothing startling will be attempted, nothing original undertaken; content if able to point out some of the well-known dangers which beset the beaten path of life, and furnish a few plain rules by which to regulate your conduct towards your professional brethren, your patients, and society.

The first question claiming attention from the neophite, where shall our lot be cast? is of undoubted importance. The *first* location should be the only one. No greater error can be committed by the young practitioner, than by supposing it easy to change to a better, if not satisfied with that first adopted, and thus select a small town with the expectation of subsequently removing to a larger. The physician's capital consists almost wholly of reputation, of the good opinion of those who believe they have been relieved by his ministrations. It is, therefore, local in character, and cannot be transferred. He who changes his place of residence late in life, no matter how high his position may have been in the community where he has already labored fully; having lost by age the aptitude for acquiring and the patience to wait for business, is doomed to disappointment. This first step, therefore, as its results are life-long in duration, should not be inconsiderately taken.

In Russia and some parts of Germany, government restricts the number of medical men. In Bavaria, particularly, there is a limitation to the number of practitioners, and no person is allowed to commence practice until it is ascertained that there is a vacancy for him. The inspector of each district, having a list of all the doctors under his jurisdiction, upon the death of any one of them, fills his place from among the young candidates for service. In this selection he is said to be governed often more by political influences than the merits of the applicants, taking those only whose opinions are agreeable to government. In this way, you perceive, an excess of medical practitioners is effectually prevented; but producing as the inevitable result of this system, a class of mechanical students, utterly destitute of moral and intellectual attainments, and who after their appointment sink into contented mediocrity.

Great as are the evils which sometimes attend competition in this country, I confess I should be very sorry indeed to see any other restraint laid upon the increase of medical men, than the wholesome check of increased requirements, and more thorough education. No, confiding in your own determination to be useful, and to ensure success by your real merits, go to that place where such climate, society, social and religious institutions as you think will most contribnte to your own happiness, are to be found; where, when business is obtained it will be worth possessing; where the seed sown iu youth when it ripens shall bear fruit worth the gathering in maturity, and where old age can find its solace, and thank Heaven that its lot is "cast in pleasant places." Select, I say, such a location, and then set vigorously at work preparing for the responsibilities attending the discharge of professional duties, never doubting that success will attend your efforts.

This resolution formed, and the young physician, having established himself in the place where he is to remain, should immediately commence to study the various peculiarities of climate, natural influences, tendencies of the prevailing occupations and habits of life of the inhabitants of the place of his adoption. Bear in mind that in proportion as it is perceived that all the circumstances of life, both moral and physical, may be employed for the preservation of health, medical science approaches the problem of giving laws to life. In your subsequent intercourse with your patients you will thus be enabled to lead them to form just views on important questions of hygiene. Lose no opportunity of urging them, and the authorities having charge of the sanitary condition of the place, not to disregard established natural laws, but to look to prevention as well as cure. Few physicians, unless their attention has been especially directed to the subject, can have any idea of the amount of ignorance which exists, even amongst the educated classes, on these subjects. Persons may be found in this and almost every other community, who have the pecuniary ability to choose, who nevertheless live for years, sometimes their whole lives, in houses where imperfect drainage, the absence of proper ventilation, or exposure to miasmatic influences, keep up a constant succession of invalids in their family, spending yearly large sums in medical attendance, and constantly wondering why they are so sickly.

Still further:—Philanthropy and your own health, and the health of your family; for it is not less essential to the physician than to every other member of community, that the healthfulness of the locality in which he resides should be improved to the greatest possible degree; philanthropy and self-interest concur, I say, in inducing you to ascertain and make known the causes of any endemic or epidemic diseases whieh prevail in your neighborhood. Nor will this enlightened study and advocacy of the cause of humanity in which the poor have so large an interest be without its reward. It is the best way of bringing yourselves usefully before the public, and that too in the legitimate sphere of professional duty. Humanity in the widest signification of the term, is the object which is assigned to the medical profession; its inalienable qualification is to be cautious, indulgent, and in all the relations of life to be helpful to others in advice and action. In the labyrinth of disease you must search for and hold fast to the clue of its origin, connexions and course; this is the thread of Ariadne which will certainly conduct you to the goal of success. The more intricate the case at first appears, the more earnestly must you labor to separate the essential from the non-essential, and to discover the real cause of any prevailing disease. These laudable efforts will be highly appreciated in every community. Nay, more, they will serve to make you thoroughly versed in the *science* of medicine, which is the only sure foundation upon which a medical reputation can be built. It is true, a happy concurrence of circumstances, or family influence may raise a man destitute of solid attainments to an elevation above his merits; but his position is ever insecure, and a few years will find him sunk in forgotten insignificance.

Do not allow yourselves to become political partisans. Never run the hazard of having your duties to patients, who have entrusted their lives to your care, interfered with by political engagements. Your studies, habits, and occupations unfit you for the discharge of the duties devolving upon the recipient of office; and it were better you should never become a candidate for any position in the gift of a party. You cannot, by partisan aid, permanently advance your professional interests. You should, indeed, have well formed and correct opinions upon all the public measures of the day, and in casting your vote it will be well for you, keeping aloof from party tramels, to give it such direction as to lessen political acerbity, and exert a conservative influence. Do not, however, for one moment, suppose that I desire to see you converted into such neutral mixtures that you will hesitate in the recognition and discharge of your whole duty when the liberties of the country are imperilled. No, in addition to the fearless expression of patriotic opinions, you should be found at your post in the camp and in the hospital. Nobly were these self-sacrificing labors performed by the surgeons of America throughout the fearful struggle from which the country is just emerging. Many established practitioners exchanged the comforts of home, liberal incomes, and honorable positions in society, for the less remunerative service of the government and the privations of the camp.

From a very extensive intercourse with the members of the profession on the border between the two contending sections, in the execution of commissions which brought me into intimate relations with them all the way from Fortress Monroe on the east to Memphis in the west, I am proud to say I found them, as a class, thoroughly loyal. Associated on another occasion with my friend and colleague, Prof. Rochester, in an inspection of the hospitals of the west and south, authorized by the Surgeon-General and the Sanitary Commission, I venture to affirm that he will also bear testimony to the zeal and patriotism everywhere manifested by our medical brethren. Of the heroic examples of surgeons on the field; of their personal exposures to danger; of their voluntarily submitting often to be taken prisoners, rather than forsake the men under their charge; of their sufferings from disease, unattended by medical brethren; of their unintermitting labors; of such like incidents a volume might be written. Brighter examples are not recorded on the fairest pages of history. In our own circle, witness the vacant seats often heretofore occupied on this anniversary, by the magnanimous and patriotic Wilcox; the conscientious and scholarly Washburn; the accomplished and heroic surgeon Irwin; and the youthful and zealous Butler. These men deserve the commendation of their countrymen. Their examples are worthy your imitation!

As a substitute for political excitement and occupation, permit me to urge you to prosecute hereafter your professional reading. Think not that your education is now complete. You have but made that amount of progress in the various departments of medical science; you have but gained that degree of intellectual strength which will enable you to pursue your investigations unaided by instruction. You can now grope your own way into the light, though yet tottering and uncertain in your step. Should you flatter yourselves that hereafter study is unnecessary, you will strand upon the bar which lies at the entrance of the harbor, before the voyage is fairly commenced. Conceit cannot long endure as the substitute for solid attainment. It is a well known law in capillary attraction, that fluids stand highest in the smallest tubes; so the ignorant, empty-headed, narrow-minded physician, too wise to learn, will often be found assuming the most lofty bearing. And as certainly as these same liquids lose their elevation when deprived of artificial support, so those men of small caliber and large pretensions will find their level, when brought into intimate relations with other members of the profession without their adventitious prop.

Not only must you continue to study, but in order that you make true progress it is of great importance that you prosecute these studies aright. It is a fact not sufficiently understood and acted upon, that education is of two kinds. There is, first, the education of the mind—that training and culture of the mental faculties—of the judgment—the reasoning powers—the taste and appreciation of those subjects which shall afterwards be presented, and which fits the mind for the purposes to which it is destined. Secondly, there is that special education of the mind thus disciplined and fitted for that particular employment in which it is henceforth to be engaged. The former of these departments of education is by far the most important, and it is precisely that in which our present system of medical education—teaching by demonstrative lectures—is most deficient. It is because undue importance is attached to this second department in education—the acquirement of the special knowledge required for actual use, that classical studies have been unduly depreciated in the education of medical men. It is true they are not in reality medical studies; but they discipline and enrich the mind, and prepare it for the study of the sciences; and they have a counteracting influence to the purely material atmosphere with which the absorption in physical science envelopes the mind of the medical student.

It is the *mind*, then, I repeat—the *mind* which should be the first object of attention—not the mere acquisition of knowledge, but the cultivation of that instrument by which knowledge is acquired, and by which alone that knowledge can be made available. The human mind, says an eminent author, may fitly be called "that great and universal machine, by which we operate upon all things." In improving, then, this universal engine, we are conferring a service on mankind something the same in kind with the improvement of the steam engine; but in degree and extent of usefulness beyond all comparison greater. If this be important with regard to mind in general, how much more so with regard to those minds which are to be exercised upon a profession like medicine?

Time was when those practicing the healing art possessed *all* the learning, hording it as a legacy peculiarly their own. The Gospel of Luke, the physician, is said to be written in purer Greek than any of the other Gospels; and among the moderns, medicine can claim some of the brightest stars in literature and poetry. Away then, with this modern under-estimate of the value of classical and literary attainments to the medical student! Not that you should attempt everything, and so fail in all. For it is on the contrary true, that this is an age of ambitious acquirement, and some professional men seem to be ashamed unless they have the character of universal knowledge. He who falls into the error of studying everything will be certain to know nothing well. Disregard the clamor of the age in which we live, also in favor of general education. Avoid the yellow covered literature, the trashy periodicals, the flashy, popular lectures, with which we are at the present day assailed by the ceaseless competition of those who vend cheap knowledge under these various and tempting forms. Strive rather for that calm and unpretending acquirement and secure precise study, without which the effort to become good physicians and surgeons must prove vain and fruitless.

A most useful practice in the accomplishment of this end consists in carefully recording such cases daily as possess interest, with such comments as are at the time suggested. Perhaps no exercise combines mental discipline to a greater degree, at the same time that it serves to keep the mental faculties actively engaged with the subjects peculiarly belonging to the practitioner. Indeed, it may with truth be said, that the most instructive history to the physician is the history of disease, where every circumstance pertaining to its origin, progress and termination as it transpires, has been carefully noted by himself. In this way only can you come to the knowledge of yourselves and your own views of disease. Know ourselves? do I hear you enquire? Do you not remember that at the entrance of the Delphic Apollo, the most famous oracle of ancient Greece, which for a long time enjoyed the reputation of infallibility, there were inscribed in golden letters, "Know thyself?" plainly intimating to its votaries the source of true Pythian inspiration.

Many, doubtless, will excuse themselves from making this historic record of cases for want of time. It is unquestionably true, that the active duties of professional life leave but little time for medical writing, and it requires considerable self-sacrifice to sit down to the desk after the labors of the day are over. But if time be economized, scarcely will a day be so completely occupied as not to leave space for the fulfillment of this duty. Many bright examples might be cited in support of this assertion. Two only will be mentioned.

Prof. (Sir James) Simpson, of Edinburgh, and Marshall Hall, of London, the most original and useful contributors within the last quarter of a century, to the two most important practical departments, Obstetrics and Practic of Medicine, have personally assured me, that the entire manuscript for their monographs was written whilst in their carriages going from patient to patient. Those whose position and extensive opportunities for observation enable them to make the most valuable contributions to medical science are precisely the men who are under the strongest temptations to neglect the effort. Disinclination, or neglect of its possessors to put in written form the result of their valuable observations, has lost them to the profession and mankind, and the fruits of years of arduous labor and careful observation has been buried with them in the tomb.

In order to render this record or history useful to yourselves or others, truthfulness is an indispensable qualification. Otherwise this chronicle becomes by its incorrectness a slander of long continuance, and like the poison tree in which most of the constituent parts resemble the innocent plants which surround it, whilst it bears concealed in its flower that which stamps it as the fatal upas. Again there should be minutely and distinctly enumerated all those attendant circumstances which accompany the progress of disease and modify its character, else they lose all their individuality, interest, and value. Half the truth is often a lie, and no where is this more frequently the case than in medical narrations. Unintentional errors of this kind are often committed by men of sanguine temperament, with a darling theory to sustain. In the advocacy of some new plan of treatment which they have adopted, they collect together a number of cases loosely recorded, and which, though not really, seem to them similar in character, and which they promulgate in order to prove the efficacy of their peculiar plan. Our medical journals abound in cases of this description. The first essentials, then, we repeat, are correctness, accuracy, and fullness of detail. When conducted in accordance with these rules this practice will be found the best remedy for a bad memory; it will sharpen the intellect, teach the writer the art of questioning disease, and lead him to correct conclusions. Having recorded a series of symptoms, he is naturally led to inquire whence each arises; his attention is attracted to anything unusual, and he learns to question nature and to deduce order from her variableness, and truth from the multitude of conflicting appearances. With one other suggestion we will dismiss this important subject. Let every one adopt, in recording their experience, the system known as the numerical method; it is essential to the attainment of the ends proposed.

Again, those of you who have been most industrious and who have brought to the labor minds well prepared by previous discipline for the task, have found the period of study too brief for acquiring more than the rudiments of a medical education. It is true you have quited yourselves like men in the ordeal to which you have been subjected. You are now ready to undertake the investigation of numerous topics necessarily omitted during your pupilage for want of time. The school has made the beginning, and you are now prepared for independent effort. Not only must you study the past and present, but keep pace with the progress which is constantly making in the various departments of medical science. Else will you be left far behind in the race; and, like drift-wood, which, forsaking the current upon which it was floated rapidly forward, seeks the stagnant pool at the river-side, and not only becomes stationary itself, but entangles other passing timbers until by its accumulation it chokes the stream and greatly retards the progress of all who may navigate its waters. Medicine is ever extending. Journeys into new parts of the world enlarge the materia medica. Chemistry is constantly supplying the laboratory not only with new substances, but new combinations of old are daily formed, enlarging the boundaries of the pharmacopæia. The microscope, though but yesterday made subservient to the interests of medical science, now daily brings to light new facts which directly concern the physiologist and pathologist, and this instrument should now be found upon the table of every practitioner who hopes to discharge his whole duty to his patients. Indeed, gentlemen, in London, which at this moment is taking the lead in the race of true progress in medical science, I found during a recent visit that to resort to the microscope in diagnosis, was as much a matter of course as to the watch in numbering the frequency of the pulse. I am aware that there are not wanting those in every neighborhood, who ridicule its use as an auxiliary in diagnosis; who never lose an opportunity of hinting to the people that those who love the *science* of medicine are not *practical* men, though they may be very learned. Too indolent to keep pace with improvement themselves, they depreciate all progress, and abuse its advocates. They are the fossilized, faded monuments of the obsolete doctrines and theories which prevailed when they "were educated." Would that they had been *educated*—that their minds had received that discipline already described, which makes scientific investigations attractive, and would have enabled them to appreciate the value of the real improvements which have been made in the practice of medicine within the last quarter of a century since they left school, or as they claim, "*finished* their education." Many of the theories of that day are long since exploded, and with all intelligent men of the present, have become historic. Such men become mere routinists, and may be found in every community. They might appropriately be called bleeding and blistering machines. They go from house to house, leaving with every successive patient the same unvarying dose of calomel and jalap—exercising as little intellectual effort at a rational analysis of the symptoms presented as a planting machine, which at each revolution of its clumsy wheel, deposits a uniform quantity of seed. More than this; to conceal their own ignorance and indolence, they sagely shake their heads and warn community to beware of all men of progress, who reduce the art of prescribing to a rational process—and by the aid of scientific rules, after a careful investigation of each malady, govern their therapeutical recommendations by the light of modern pathology. With envious finger do those sluggards point to all such as innovators, introducers of foreign notions, and as destitute of true practical wisdom. They importune the very mouths which they have made sore and disfigured by unnecessary visitations from their mercurial deity, to join in their anathemas against all advocates of rational reform and improvement.

Is it a matter of surprise that to escape these thoughtless mechanical routinists, intelligent men so often seek refuge in quackery? Here may be found the true "*pabulum vitæ*" of the various "isms" of the day, science itself being often held responsible for the stupidity of those who wear its livery. Would you avoid imitating their example and not dishonor the profession of your choice? If so, cherish the habits of the student, and mark the progress constantly making in the various departments of medical science.

But whilst you avoid Scylla fall not into Charybdis. Do not adopt every untried novelty with which the medical press is daily teeming. Nor jeopardize the lives of your patients by implicit obedience to foreign authority. The visionary, and he alone, thinks the distant country as beautiful as it appears when enveloped in blue ether; submit all new suggestions to a careful examination; place them in the crucible of observation, and if truth be elicited, add it to the stock already at disposal. It is far better to make solid, though humble additions, than to obtain an evanescent notoriety as the propounder of new doctrines. Unless guarded against nothing is more likely to occur with young men of sanguine temperament than the adoption of theories or riding of hobbies. This is fatal to success. The practitioner, tainted with this mental obliquity, approaching the bed-side with a foregone conclusion to confirm and sustain, is no more likely to judge correctly of the indications presented, than he who regards an object only by light transmitted through stained media can determine its peculiar shade or hue.

Sometimes a system of magnifying the merits of plans of treatment claimed to be peculiar, is carried so far and urged upon popular favor with so little scruple, as to deserve to be branded as empiricism. Thus, when a man for the sake of gaining practice takes up the fashionable folly of the day, or adopts some peculiar plan of treatment, not because convinced of its utility, but for the sake of producing a sensation in the public mind, and making himself notorious. Such men might have been seen abusing a good cause, by going about the streets with a stethescope projecting from the pocket when auscultation was first received into general favor.

Again, the publication of works in a popular form, or articles in the secular journals recommending some plan of treatment which can only be employed by the author himself, is deserving of still greater reprehension. It is certainly a fair and legitimate line of conduct for a young man to write and publish with a view to introducing himself to notice; but let him seek to obtain this by means of what is really true and valuable, worked out by study and experience, not by some novel and specious doctrines which can merely serve to catch the suffrages of the unthinking. There are many degrees of this kind of quackery, but all of them are utterly unworthy of an ingenuous, honest mind, derogatory to the character of a man of learning and observation, and tend to lower the practice of medicine as a science.

Against the open advocacy of charlatanry, when the medical practitioner outraging conscience, and judgment, and integrity, avowedly attempts to live upon the credulity of the public, a line of conduct so obviously unprincipled, that he who practices it at once loses the confidence of his colleagues, and is virtually expelled from the ranks of the profession; against the adoption of such a course it is unnecessary to caution you.

Believing it a waste of your time, I shall pass unnoticed the quackery practiced by non-medical persons, merely as a speculation or trade, selling panaceas, and the various ephemeral "opathies" which follow each other in rapid succession. There is a class of minds in every community who must ever be dealing in the marvellous, and would scarcely credit our disinterestedness should we raise the voice of warning. Better not confirm them in their heresy by provoking them to fortify themselves with fictitious arguments to support their false positions. All contentions with quacks and their partisans should, in my judgment, be scrupulously avoided, as at best but a fruitless waste of time, and often giving notoriety and importance to doctrines which otherwise would attract little attention.

Permit me in the next place to say a few words relative to your deportment towards your professional brethren. Let it be remembered that medical men may always claim for themselves and families the gratuitous attendance of their brethren, whatever be their several pecuniary conditions. Where expense in traveling is incurred, that of course should be defrayed by the party benefited. This is a well established rule in medical ethics, and requires only to be mentioned that it be adopted. Let all your intercourse with your professional brethren be distinguished by candor and liberality, and the total absence of professional trick. In consultations ever bear in mind that personal animosities, if they exist, must be dismissed, and nothing should be had in view but the welfare of the patient. Many advantages may arise from consultations among practitioners, where men of candor, who have mutual confidence in each other's honor, unite for a common purpose. A remedy may occur to one which did not to another, and a physician may want resolution, or sufficient confidence in his own opinion, to prescribe a powerful but precarious remedy, on which, however, the life of his patient may depend; in this, and analagous cases, a concurring opinion may serve to establish his own. But where mutual confidence is wanting, where opinions are regarded, not according to their intrinsic merit, but according to the person from whom they proceed; or where there is reason to believe that sentiments delivered with openness are to be whispered abroad and misrepresented to the public, without regard to the obligations of honor and secrecy, in such cases consultations of physicians tend rather to the detriment than the advantage of the sick, and their ordinary conclusion is a compromise, adopting some very harmless but insignificant prescription. There are also duties in consultation, not only in reference to the patient, but in reference to those composing it amongst themselves. What can be more cruel than for a senior practitioner to treat his younger brother with slight and superciliousness? instead of encouraging him in his arduous duties. What more mean than to withhold from him his just meed of praise, or by insinuation as to his course to lower him in his patient's estimation and lessen his confidence in him for the future! There is one great principle in medical ethics which should never be deviated from, in consultations among physicians; that of doing as much good as possible to the patient with as little injury as possible to those previously in attendance. Differences of opinion should never escape the precincts of the consulting room. That reputation can alone be solid or lasting which is based on the adjudication of the only legitimate tribunal—the voice of the profession—and whose favorable verdict is far more to be coveted than all other earthly possessions.

Of all the causes which tend to lower the medical profession in public esteem, the suspicious jealousy of medical men, their want of self-respect and a right understanding amongst themselves, amounting often to declared hostility, is the most effective. The quarrels of physicians, when they end in appeals to the public, injure not only the contending parties, but throw discredit upon the whole profession, and expose the faculty to ridicule and contempt. When you come to practice be most careful, therefore, to shun the habit of depreciating other practitioners. The reflection which invariably flits through the mind of the hearer, is, that your remarks are dictated by envy or jealousy, and thus you not only degrade yourself but the profession. He who indulges in this pernicious habit can have no fine perceptions of the principle of justice.

As a means of preventing these lamentable differences among doctors every opportunity for meeting for scientific and social intercourse should be embraced. No antidote is more effectual in curing unfounded prejudice towards those who possess any real merit than frequent interviews for social intercourse or interchange of sentiment upon topics of mutual interest. It is nevertheless true, that the only infallible remedy for the hydra-headed evil of medical jealousy, consists in the elevation of view and purity of purpose of the individuals themselves. Indeed your motives will challenge suspicion if detraction of a fellow practitioner be indulged whilst engaged in the discharge of professional duties, for malice should be wholly unknown to the physician who only desires to render assistance to his patients. The wounds inflicted by laymen to our reputation are simple, and easily healed—incised wounds; on the contrary those inflicted by medical brethren upon each other are lacerated wounds—they close slowly and leave awkward, unseemly scars behind. Shun, therefore, a gossiping doctor, and take the same pains to arrest the circulation of the false reports against medical men which he may have originated as you would the circulation of spurious coin. Against unjust censure from the community to which the medical man is peculiarly exposed the best remedy is patience, knowing that merit always outlives calumny, striving meanwhile by cultivating the highest motives, to live above the influence of these attacks. Much may be done to lessen the groundless censure of the community by gradually enlightening the public mind on medical subjects; teach men correctly to estimate what may and what may not be expected of medicine; increase the sense of the impropriety of positive and rash judgment on matters at once so delicate and so difficult of decision. None but those who are in the profession, or who may have been admitted into the most secret confidence of a medical man can tell the anxieties which attend him. In every step he takes his character is pledged; and so delicate is the nature of a good name, so easily is it tarnished that one rash act may forever demolish it, whilst it cannot be much enhanced in brilliancy by a judicious one. Who can tell the prudence and foresight which must belong to every successful physician? Who can estimate the anxieties which disturb his peace? Let the profession be united then in sustaining each other as far as truth and justice will permit, and let every member as he beholds the esculapian serpent coiled around his staff as the symbol of rejuvanescence, be reminded also how often the physician has to deal with the coldness and poisonous fangs of mankind, and be incited to professional unity.

Whilst claiming your sympathies for the whole medical profession and your hearty coöperation in promoting harmony among its members, permit me to bespeak a kindly interest for your *Alma Mater*. No matter in what direction duty or inclination may lead you in the prosecution of your professional labors, doubt not that your course and its results will be objects of intense solicitude to all for whom I this night address you. Every laurel which you may win will be a source of pride to those who have aided you in the preparation for the conflict, and all your successes will be claimed as reflecting honor upon the college whose maternity I trust it will ever be your boast to claim. Indulge me just here with one word of history.—A little more than twenty years have elapsed since Dr. Austin Flint and the speaker, aided by a few friends, procured, through the influence of Hon. N. K. Hall, then in the State Senate, the charter for the University of Buffalo. Notwithstanding the apprehensions of the friends of the enterprise, and the ridicule of its enemies, we proceeded immediately to organize the medical department and appoint its faculty. A building was rented and fitted up for temporary use, and the work of teaching entered upon with energy and zeal. Our success more than equalled the expectations of the most sanguine, and within a short period, unaided by the bounty of the State, which had been generously extended to all the other medical colleges, by the liberality of our citizens we were enabled to erect a substantial structure for our permanent accommodation. Thus began the first permanently successful effort to establish, in this great commercial city, an educational institution above the grade of the common school. We were able at once to transfer most of the members composing the faculty of the medical college of Geneva to Buffalo. Profs. C. B. Coventry, of Utica; Charles A. Lee, of New York, and James Webster, of Rochester, were thus transferred. Frank H. Hamilton, then and since distinguished as a writer and teacher in surgery, also at the time a member of the Geneva faculty, immediately embarked in the new enterprise, having already come to Buffalo to reside. It immediately assumed a position among the first schools of the country, and its chairs have ever been filled by men of learning and ability. Dr. Flint, my co-laborer in the initiation of this undertaking, has achieved a world-wide reputation as a writer and teacher in his department, and has, in my opinion, within the last year, published the best work on the "Practice of Medicine," to be found in the English language. In physiology, we claim to have been the first in this country to introduce experimental teaching. In this institution were brought forward besides the present popular incumbent of the chair, the two ablest American writers and teachers in that department now living. With the present faculty you are thoroughly acquainted, having listened to their instructions during the last session, and I do not doubt fully appreciate their merits, and it cannot therefore be necessary for me to dwell upon their claims to your consideration.

On the part of this institution be assured that it has ever been the deliberate determination of the faculty of this University to maintain a position second to none in the country. In many things essential to an elevated standard of medical education the founders of this college claim to have led the way in reform and improvement. It has ever been also the desire of those to whom the interests of this institution have been confided to send forth such Alumni only as would do honor to that profession to which its credentials might form the passport. As an evidence of the thoroughness of the course of instruction in this institution, the faculty are gratified in being able to assert that, among all the candidates who presented themselves during the war for examination before the medical boards of the different States; or the more exacting United States Army Board; or the still more severe scrutiny of the medical staff for the Navy, they have yet to learn the first rejection of one holding its degree of Doctor in Medicine. We prefer to be judged by the quality rather than the amount of fruit annually furnished, relying upon the ultimate and permanent attainment of success in this way, rather than temporarily enjoy the ephemeral reputation of cheap lectures and indiscriminate graduations. With pride can we point to a long list of honorable names as the representatives of these principles, and with the Roman matron exclaim, "these are our jewels." Convinced that by increasing the facilities for a more thorough and efficient course of instruction, as well as by making a high standard of attainment requisite to the reception of its honors the true interests of the student and the school are alike consulted, our course now as heretofore shall be onward and upward. Undismayed by opposition which may temporarily threaten but cannot seriously embarrass, never turning aside to solicit patronage or redress our wrongs, our business shall continue to consist in raising higher and still higher the claims of its several departments to the consideration of the profession. Actuated by such motives, influenced by such resolves, with the present efficient and able organization, by industriously persevering in the course already so successfully pursued, shall we not bear aloft the reputation of this institution, and place it on an elevated and permanent basis? Yes, gentlemen, our star is already in the ascendant—our success already assured.

But time fails me. I had hoped to be able to say a few words in relation to your duty to the sick poor; to beg you to discriminate between true and false benevolence; to add some words of encouragement in the discharge of your arduous and responsible duties, and of warning against yielding to despair at the loss of patients, and to touch upon some other kindred topics, but I forbear.

In conclusion, gentlemen, at the same time that I urge upon you faithfully to pursue the study and the practice of the several departments of your profession, I could wish it were in my power also to set before you the ease with which its practice is pursued and the liberality of its rewards. On the contrary I am constrained to admit that the whole business of a medical man is of such a nature as to excite the commiseration of persons employed in other pursuits less imperative and capricious in their demands. The Sabbath is seldom to him a day of rest, night does not always secure cessation from labor, nor the storm find him sheltered. The pangs of disease and the agonies of death recognize no distinction of days or seasons, no claims of society or kindred; and they who require our services look upon us as soldiers on duty, subject to their calls at all times and under all circumstances; and they expect us to render a cheerful compliance to their demands, no matter how untimely or unreasonable. Nor as business may increase with advancing years and reputation, can you hope to lessen your labors by having a part of your duties discharged by substitute. Yet, for all these sacrifices of self, neglect of family, and his own comforts, pleasures and interests, is the pecuniary compensation of the physician most beggarly. Sometimes a life of great labor and privation, affording scarcely sufficient revenue to provide for his family, and respectably educate his children.

Fortunately, gentlemen, our reward does not consist entirely in the pecuniary compensation—it is not merely a mercenary trade which we pursue. Be induced to take Him for your exemplar, "who went about doing good," and make your chief happiness to consist in the consciousness that your aim and purpose are the amelioration of human suffering. And though I cannot promise you pecuniary riches or luxurious ease, I have no hesitation in saying that a far richer reward awaits you, in an approving consciousness of duties deliberately assumed and honestly discharged; in the gratitude of those whose lives you have saved, or whose sufferings you have alleviated; or who regard you as the happy instrument in the hands of an overruling Providence of preserving the lives of offspring more precious than their own; and if pursued with right motives and a religious trust as your last and greatest reward, a fadeless inheritance.

